# Telerehabilitation in Multiple Sclerosis: Results of a Randomized Feasibility and Efficacy Pilot Study

**DOI:** 10.5195/ijt.2018.6256

**Published:** 2018-12-11

**Authors:** CECILIE FJELDSTAD-PARDO, AMY THIESSEN, GABRIEL PARDO

**Affiliations:** OKLAHOMA MEDICAL RESEARCH FOUNDATION, MULTIPLE SCLEROSIS CENTER OF EXCELLENCE, OKLAHOMA CITY, OKLAHOMA, USA

**Keywords:** Balance, Gait, Multiple Sclerosis, Telerehabilitation

## Abstract

A prospective, randomized, three-arm, evaluator blinded study to demonstrate the feasibility of a telerehabilitation (TR) program in individuals with ambulatory deficits secondary to Multiple Sclerosis (MS) and evaluate its efficacy when compared to conventional on-site physical therapy (PT) was completed. Thirty participants were evaluated at baseline and randomized to one of three groups with intervention lasting 8 weeks: Group 1 (control)- customized unsupervised home-based exercise program (HEP) 5 days a week; Group 2 (TR)- remote PT supervised via audio/visual real-time telecommunication twice weekly; Group 3 (PT)- in-person PT at the medical facility twice weekly. Outcomes included patient reported outcomes (PROs) obtained through questionnaires, and measurements of gait and balance performed with bedside tests and a computerized system. Functional gait assessment improved from baseline in all three groups. There were no significant differences between the TR and the conventional PT groups for a variety of outcome measures. TR is a feasible method to perform PT in persons with MS and has comparable efficacy to conventional in-person PT as measured by patient reported outcomes and objective outcomes of gait and balance.

Gait dysfunction has been identified by persons with multiple sclerosis (MS) as the most concerning limitation (Cameron & Wagner, 2011; Heesen et al., 2008), and is a common manifestation with surveys establishing that 41% have ambulatory deficits and 54% experience imbalance (Larocca, 2011). Decreased postural balance has been suggested as the leading cause of falls in people with MS with 52% of participants reporting a fall in the past 6 months (Finlayson, Peterson, & Cho, 2006). Nilsagard, Lundholm, Denison, and Gunnarsson (2009) reported 63% of studied MS participants fell at least once during a 3-month period with an increase in frequency conditioned by aging and disease progression. The risk or the fear of falling affects these individuals’ participation in social interactions and physical activities and can lead to a negative effect on their physical and emotional independence.

Functional improvement of established physical deficits can be achieved through different interventions to include neurorehabilitation methods such as physical therapy (PT). These treatments aim to reduce existing disabilities and increase functional independence. Optimizing the functional ambulatory status of people with MS could result in improved quality of life, independence, and safety.

PT services are delivered in outpatient settings at hospitals and specialized clinics, or at home through home health services. Access and adherence to specialized PT interventions are limited by a variety of factors such as availability, geographical location, mobility limitations, time constraint, transportation difficulties, health insurance coverage, and financial burden (Petajan & White, 1999; Rio et al., 2005). Addressing these barriers is an important and necessary step in improving patient care in MS.

Telecommunication technology offers the capacity to supervise and direct a PT program remotely through audio and visual real-time communication and is a viable solution to minimize several of the identified barriers to care. Given the variety of factors that impair access to specialized rehabilitation services in MS, designing and implementing a telecommunication PT program would provide a practical, accessible, and effective way to improve function and well-being. In addition, performing the program in the home setting could facilitate adherence, adapt to the real life environment, improve self-reliability, and generate a therapeutic alliance with the caregiver.

Although telerehabilitation (TR) research is still in its early stages, preliminary studies have shown some improvement in balance and postural control in people with MS that underwent a TR program (Gutierrez et al., 2013; Ortiz-Gutierrez et al., 2013). No adverse events have been identified as a consequence of utilizing this intervention (Khan, Amatya, Kesselring, & Galea, 2015). A study by Finkelstein, Lapshin, Castro, Cha, and Provance (2008) implemented a 12 week physical TR program for individuals with MS that resulted in significant improvement of the 25-foot walk, 6-minute walk and the Berg Balance Scale (BBS) tests compared to baselines scores.

## METHODS

A proof of concept prospective, randomized, three-arm, evaluator blinded, 8-week pilot study with 30 subjects randomized in a 1:1:1 fashion was conducted. All individuals underwent a baseline medical and PT evaluation by a neurologist with expertise in MS (GP) and a physical therapist with extensive knowledge of this condition (AT) respectively. Participants were assigned to an unsupervised customized exercise program, or to supervised adaptable sessions with the treating physical therapist either through telecommunication or in-person, all lasting eight weeks, resulting in the three study groups: Group 1- unsupervised HEP (control group) five days a week; Group 2- remote PT supervised via audio/visual real-time telecommunication twice weekly (TR group); Group 3- HEP plus in-person PT at the Oklahoma Medical Research Foundation (OMRF) Multiple Sclerosis Center of Excellence PT facility two times weekly (PT group). One patient in the PT group dropped out due to an MS relapse.

The OMRF institutional review board approved the study. The logistics of the telecommunication process were optimized with full compliance with privacy regulations. All participants had to sign an informed consent prior to participating in the study. There were no barriers identified for participation in the TR group.

Outcome variables included clinical assessments of gait and balance and were obtained at baseline and at end of study by a single evaluator (CF-P) who was blinded to the group allocation. Gait and balance variables were measured using the NeuroCom Smart Balance Master (Natus Medical Incorporated, Pleasanton, CA) at baseline and exit visits. This system has demonstrated good utility for evaluating gait and balance in MS (Fjeldstad, Pardo, Bemben, & Bemben, 2011) and other diseases (Burke, Franca, Ferreira de Meneses, Cardoso, & Marques, 2010; Ondo et al., 2000). The device is equipped with a movable visual surround and a dual-plate force platform with capability of rotation in order to measure vertical forces exerted by the participant’s feet on the force plate during testing. Further, the long force plate component evaluates walking and postural control during ambulation.

### GAIT MEASURES CONDUCTED

#### FUNCTIONAL GAIT ASSESSMENT

Functional gait assessment (FGA) is a 10-item evaluation of gait function. Each item ranges from 0 (severe impairment) to 3 (normal). A maximum score of 30 is possible. A 6-meter (20-foot) walkway marked with a 30.48 cm (12 inch) width is required for this test. Tests include gait on level surface, change in gait speed, gait with horizontal head turn, gait with vertical head turn, gait with pivot turn, step over obstacle, gait with narrow base of support, gait with eyes closed, ambulating backwards, and walking a set of steps (Walker et al., 2007).

#### TIMED 25 FOOT WALK

Timed 25 foot walk (T25FW) is a quantitative mobility and leg function performance test based on a timed 25-walk. The subject is directed to one end of a clearly marked 25-foot course and instructed to walk 25 feet as quickly and safely possible. The time is recorded in seconds from the moment the first foot crosses the 0 foot mark and ends when the lead foot crosses the 25 foot mark. Participants should have a minimum 3-step start so not to begin in an idle state.

#### WALK ACROSS

Walk across (WA) quantifies characteristics of gait as the patient walks across the length of the force plate using the Neurocom Smart Balance Master. This test characterizes steady gait by having the patient begin three steps behind and continuing beyond the force plate. Parameters measured are average step width (cm) and step length (seconds).

### BALANCE MEASURES CONDUCTED

#### BERG BALANCE SCALE

Berg Balance Scale (BBS) is a widely used clinical functional test of a person’s static and dynamic balance abilities (Berg, Wood-Dauphinee, Williams, & Maki, 1992; Blum & Korner-Bitensky, 2008) designed to measure balance in a clinical setting. Items included are sustained static standing in a given position for a specific time, tandem stance, one-legged stance, and stance with eyes closed. Each item ranges from 0–4, with 0 indicating the lowest level of score and 4 the highest level of score and physical function with a maximum score of 56. A score of <45 indicates a greater risk for falling (Berg et al., 1992).

#### NEUROCOM SMART BALANCE MASTER TESTS

Neurocom Smart Balance Master tests included: (1) Tandem Walk where the participant walks heel to toe from one end of the force plate to the other in order to measure sway velocity (deg/sec) and sway width (cm); (2) Limits of Stability (LOS) which quantifies the maximum distance the patient can intentionally displace their center of gravity in the four cardinal directions as well as the four diagonal directions, and their ability to maintain stability while in those positions quantified for this study as percentage of directional control; and (3) Sensory Organizational Test (SOT) which objectively identifies any abnormalities of the participant’s use of the three sensory systems that assist in postural control, namely somatosensory, visual and vestibular input through a composite score calculated from evaluations delivering inaccurate information to the participant’s eyes, feet, and joints through sway referencing of the visual surround and the support surface (combination of normal, absent or swayed-reference vision and fixed or sway-referenced support).

### PATIENTS REPORTED OUTCOMES (PROS)

#### SHORT FORM 36

Short Form 36 (SF36) developed by RAND, is a self-report questionnaire widely used to assess generic measures of health-related quality of life and consists of 8 subscales and two summary scores. The subscales include physical functioning, role limitations due to physical problems, bodily pain, general health perceptions, vitality, social functioning, role-limitations due to emotional problems, and mental health. The two summary scores include physical (SF36p) and mental (SF36m) components. It takes approximately 10 minutes to administer, (Fischer et al., 1999; Marrie, Miller, Chelune & Cohen, 2003).

#### MODIFIED FATIGUE IMPACT SCALE

Modified Fatigue Impact Scale (MFIS) is a self-report questionnaire based on how fatigue impacts an individual’s life. It consists of 21 items and covers fatigue in terms of physical, cognitive, and psychosocial functioning. It takes 5–10 minutes to administer (Fisk et al. 1994).

#### MS SELF-EFFICACY QUESTIONNAIRE

The MS Self-Efficacy (MSSE) questionnaire is a self-report 14-item instrument to assess a general sense of perceived self-efficacy of coping with living with MS. It takes about 5 minutes to complete (Rigby, Domenech, Thornton, Tedman, & Young, 2003).

#### ACTIVITIES-SPECIFIC BALANCE CONFIDENCE SCALE

The Activities-specific Balance Confidence Scale (ABC Scale) indicates the level of confidence in performing various activities of ambulation without losing balance or becoming unsteady. Participants rate their confidence on the scale form 0% (no confidence) to 100% (complete confidence) for each of the 16 items that compose the questionnaire (Powell & Myers, 1995).

### DISEASE-SPECIFIC MEASURES

#### EXPANDED DISABILITY STATUS SCALE

Expanded Disability Status Scale (EDSS) is a metric widely used to measure disability in MS. Based on a complete neurological examination, seven different functional systems and ambulation are carefully scored. The EDSS is an ordinal clinical rating scale ranging from 0 (normal neurologic examination) to 10 (death due to MS) in half-point increments. The neurological examination that is needed to make the ratings can take anywhere from 15 minutes to a half-hour and is often administered by a neurologist (Kurtzke, 1983).

### STATISTICAL ANALYSES

Statistical analysis was completed using The R Project for Statistical Computing software (The R Foundation, 2016). T- test (two-tailed) was performed on the mean of the differences (after-before) for each variable grouped by treatment type to test for significant differences from 0 with the purpose to determine if each treatment had a statistically significant effect on the considered variable.

Next, false discovery rate (FDR) corrected pair-wise t tests (two-tailed) were performed to test for significant differences amongst the considered variable across treatments to determine if a particular treatment had a statistically significantly different effect on a variable than the other two treatments. With these two analyses it can be determined if (a) a particular treatment makes a significant impact on the considered variable, and (b) is one treatment significantly more impactful on a variable than the other treatments.

## RESULTS

### DEMOGRAPHICS

The characteristics of the group are as follows: female 69%, mean age 54.7±12.3 years, relapsing remitting MS (RRMS) 60%, secondary progressive MS (SPMS) 23%, and primary progressive MS (PPMS) 17%. The control group consisted of 8 with RRMS and 2 with SPMS. The TR group consisted of 4 RRMS, 3 SPMS and 3 PPMS. The PT group consisted of 5 RRMS, 3 SPMS and 1 PPMS. Mean expanded disability status scale (EDSS) was 4.3±1.1 for the entire cohort (control group 4.4, TR group 4.4, PT group 4.3). Descriptive characteristics distributed by group are found in Table 1.

### GAIT AND BALANCE OUTCOMES

**Figure 1:**
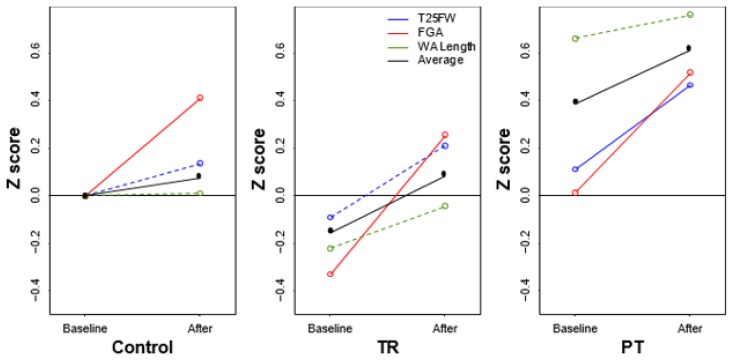
Gait scores.

**Figure 2:**
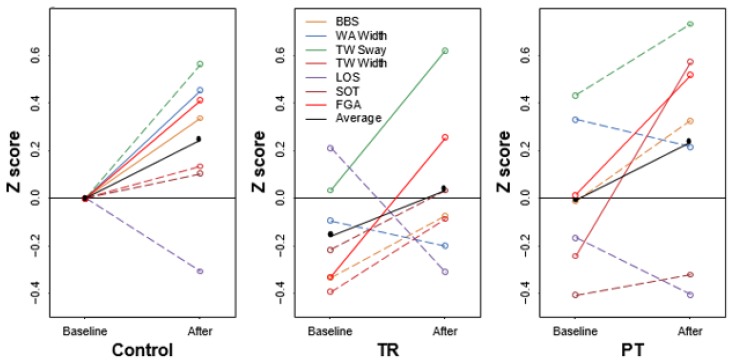
Balance scores.

The FGA improved in all three groups from baseline (P<0.05) with no significant differences between the TR and the PT group. Other outcomes that showed improvement from baseline include BBS and WA width for the control group, TW sway for the TR group, and TW width and T25FW for the PT group. Comparison of the mean differences between each pairing of groups yielded equivalent results with no statistical differences (Figures 1 and 2; Tables 2 and 3).

### PATIENT REPORTED OUTCOMES

**Figure 3:**
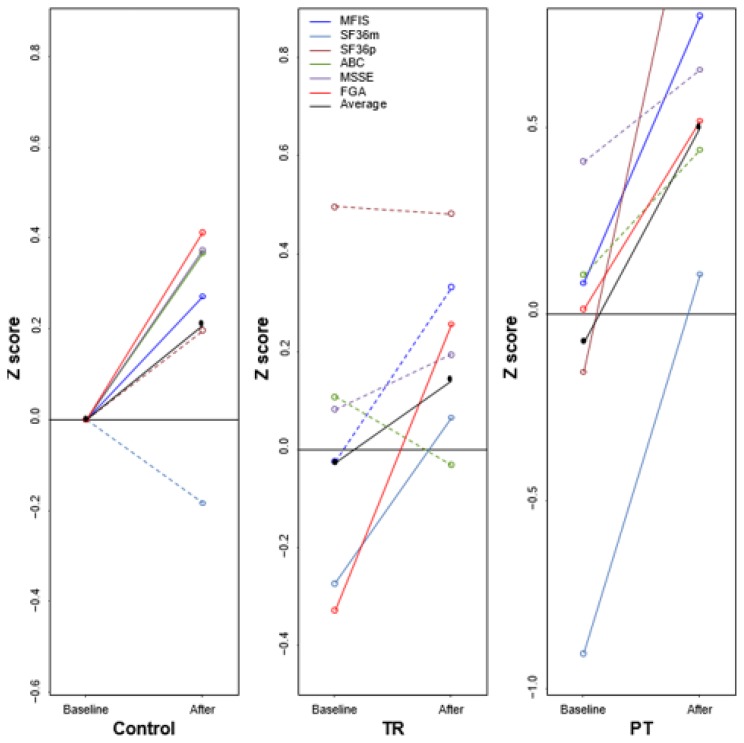
Patient reported outcomes.

The control group demonstrated significant improvement (p<0.05) in the ABC Scale, FGA, and MSSE from baseline. The TR group showed significant improvement (p<0.05) for the SF36m. The PT group had significant improvement (p<0.05) for MFIS, SF36m, and SF36p. Comparing the mean difference scores pairwise between treatment groups it was found that SF36m significantly improved in the PT group compared to the control group (p=0.0047 FDR corrected) and SF36p for PT group was significantly improved compared to the control and TR groups (p=0.0090 FDR corrected) (Figure 3 and Table 4).

For Tables 2, 3 and 4, the first three columns are the difference between the mean after treatment score minus the mean before treatment score. Columns 4–6 are the p-values for the t-tests to determine if columns 1–3 values are significantly different than 0. Columns 7–9 are the p-values (FDR corrected) for the two sample paired t-tests comparing the mean differences between each pairing of groups.

## DISCUSSION

MS can result in significant physical dysfunction, with motor impairment and decreased mobility ranking among the most common disabling symptoms (Cameron & Wagner, 2011; Heesen et al., 2008; Larocca, 2011). Access to specialized medical care is an important limiting factor in properly controlling the disease process, preventing new manifestations, and achieving maximum functional level once disability ensues. Multiple factors limit access to specialized MS care to include regional availability, geographical distance, level of physical disability, transportation logistics, employment obligations, insurance coverage and financial reasons (Petajan & White, 1999; Rio et al., 2005). Neurorehabilitation efforts directed towards regaining, improving and maintaining motor abilities can address these problems and PT is the cornerstone of such approaches. The benefits of this intervention are well documented in the literature (Giesser 2015; Motl & Pilutti 2012; Sandroff et al., 2012). Access to specialized rehabilitation professionals with knowledge of the complexity of MS is further compromised by the high number of visits that are inherent to the rehabilitation process.

Telemedicine has the capability to overcome many of the previously mentioned barriers to access to health care and provide specialized services to persons with MS. PT is conventionally performed in an individualized setting during in-person encounters between the therapist and the patient as it is traditionally considered a hands-on intervention. The possibility of using telemedicine to provide PT services is attractive and is in need of validation.

TR studies in MS have been limited but have shown encouraging results. Improvement in balance and postural control in people with MS that underwent a TR program was demonstrated when using a virtual reality system (Gutierrez et al., 2013; Ortiz-Gutierrez et al., 2013). Significant improvement in gait speed was achieved with a 12-week TR program including people with MS, (Finkelstein, Lapshin, Castro, Cha, & Provance, 2008). An internet-based study comparing TR with hippotherapy showed improvement in static and dynamic balance capacity with both interventions (Frevel & Maurer 2015). Increasing and sustaining physical activity 3 months after intervention was obtained through an internet delivered behavioral intervention in persons with MS, but no significant change in mobility or quality of life was identified (Dluglonski, Moll, Mohr, & Sandroff, 2012). Given methodological features and design characteristics, there is limited evidence to date of the efficacy of TR in improving functional activities and quality of life in adults with MS (Khan, Amatya, Kesselring, & Galea, 2015). Most these interventions, albeit delivered through a telemedicine system, were static during the duration of the trial given lack of direct interaction with a PT during the execution of the physical activity. No adverse events have been identified as a consequence of utilizing these various TR interventions.

In this study, we used a variety of outcomes related to the individual’s perception of health, fatigue, balance, and self-efficacy in addition to objective measures of gait and balance with conventional tests and novel computerized analysis systems with the objective of determining if TR had comparable results with traditional in-person PT. A unique feature was the adaptability of the TR system with modification of the exercise regimen, resembling what is done with conventional PT intervention, as each one of the remote sessions were performed live with direct audio and visual communication with the physical therapist. Furthermore, different from previous studies, the comparator groups included individuals that were undergoing in-person PT. The FGA, a main outcome of gait that assesses ambulation under a variety of conditions, improved from baseline in all three groups, to include the one performing an unsupervised, non-adaptable but customized exercise program at home. This outcome alone argues for the benefit of individualized physical activity and rehabilitation in MS. The remainder of the gait and balance outcomes either improved or remained stable. In comparing TR and PT, all of the post-intervention objective variables of ambulation were equivalent. As it pertains to PROs, the MSSE, which is a measure of self-efficacy, improved for the control group only. We speculate this result may be secondary to personal empowerment after successfully concluding a prolonged, 8-week, exercise program without direct supervision. The SF36 mental health domain improved for the TR and PT groups. Intergroup analysis between TR and PT showed a superior outcome for PT on the SF 36 physical component only.

In general, the results of the intervention with TR were comparable in effect with conventional in-person PT. There were no logistical nor health related impediments for the complete execution of the trial. Only one participant did not complete the study due to unrelated onset of an MS relapse.

Future studies should include a larger cohort with refined outcomes based on the results of this pilot study to categorically demonstrate the large-scale feasibility and effectiveness of TR. Sustained benefits should be explored with new assessments several months following the interventions. Positive results could facilitate implementation of TR as a solution for access to specialized services in remote, rural, or underserved areas, to provide rehabilitation opportunities to individuals with mobility and transportation limitations even within urban areas, and support the need for acceptance of this modality for reimbursement by third party payers.

## CONCLUSION

TR offers a feasible intervention for neurorehabilitation in persons with MS and has comparable results with conventional in-person physical therapy when measured by patient reported outcomes and objective measures of gait and balance.

## Figures and Tables

**Table 1:** Demographic Characteristics and Outcome Variables

	Total N=29	Control n=10	TR n=10	PT n=9
Age mean±SD	54.7±12.3	54.4±10.8	55.1±13.9	54.7±13.5
Female (n)	20	6	7	7
EDSSpre	4.3±1.1	4.4±1.1	4.4±1.0	4.3±1.4
EDSSpost	4.1±1.2	4.4±1.3	4.4±1.1	4.3±1.5
T25FWpre	10.0±4.7	10.0±5.8	10.5±5.2	9.5±3.5
T25FWpost	8.9±3.8	9.5±5.5	9.2±3.2	8.1±2.5
BBSpre	45.1±7.7	46.1±5.9	43.5±10.0	46.0±7.1
BBSpost	47.4±6.7	48.4±5.6	45.6±8.8	48.3±5.2
FGApre	19.0±6.5	19.8±6.1	17.6±6.5	19.8±7.7
FGApost	22.5±6.3	22.7±6.0	21.6±7.2	23.4±6.5
ABCpre	52.9±19.1	51.6±14.3	53.7±23.8	53.6±21.5
ABCpost	57.9±23.8	60.8±21.3	50.8±28.1	62.6±23.7
MSSEpre	51.0±11.9	49.1±8.5	50.1±14.7	54.1±13.0
MSSEpost	54.6±12.5	54.3±12.3	51.8±13.1	58.2±13.3
MFISpre	46.6±15.8	46.9±14.1	47.3±17.8	45.5±18.1
MFISpost	38.5±16.0	41.9±13.1	40.8±20.8	32.2±13.3
SF-36m-pre	48.1±10.6	52.6±8.6	49.3±9.3	41.9±12.5
SF-36m-post	52.4±10.9	50.5±12.0	53.3±10.3	53.8±11.8
SF-36p-pre	29.1±8.3	28.0±6.9	32.3±10.0	26.7±8.0
SF-36p-post	37.9±12.5	31.2±7.0	35.8±7.7	48.5±16.7

*Note.* Mean ± SD

**Table 2:** Gait Variables^1^

Gait	mean.control	mean.TR	mean.PT	control.diff.zero	TR.diff.zero	PT.diff.zero	p.TR-PT	p.TR-control	p.PT-
	n=10	n=10	n=9	n=10	n=10	n=9	n=10	n=10	n=9
FGA	2.9000	4.0000	3.5556	0.0002^*^	0.0006^*^	0.0095^*^	0.7308	0.7308	0.7308
T25FT	−0.5540	−1.2960	−1.3478	0.0708	0.0933	0.0255^*^	0.9566	0.6391	0.6391
WA.length	0.1400	3.1680	−0.2167	0.4841	0.0855	0.5362	0.6430	0.6430	0.9273

**Table 3:** Balance Variables

Balance	mean.control	mean.TR	mean.PT	control.diff.zero	TR.diff.zero	PT.diff.zero	p.TR-PT	p.TR-control	p.PT-control
	n=10	n=10	n=9	n=10	n=10	n=9	n=10	n=10	n=9
BBS	2.3000	2.1000	2.3333	0.0255^*^	0.0742	0.0579	0.9850	0.9850	0.9850
FGA	2.9000	4.0000	3.5556	0.0002^*^	0.0006^*^	0.0095^*^	0.7308	0.7308	0.7308
LOS	−5.9630	−9.9567	−4.8044	0.8964	0.8865	0.7257	0.9014	0.9014	0.9014
SOT	1.5000	4.0000	2.0000	0.2263	0.1737	0.3355	0.9212	0.9212	0.9212
TW.sway	−1.6780	−1.7360	−0.7922	0.0775	0.0132^*^	0.2486	0.7879	0.9658	0.7879
TW.width	−0.8820	−2.0960	−5.3733	0.2573	0.0707	0.0199^*^	0.2484	0.5918	0.1843
WA.width	−2.1260	0.5470	0.3644	0.0154^*^	0.7405	0.5837	0.9111	0.2040	0.2040

**Table 4:** Patient Reported Outcomes

PROs	mean.control	mean.TR	mean.PT	control.diff.zero	TR.diff.zero	PT.diff.zero	p.TR-PT	p.TR-control	p.PT-control
	n=10	n=10	n=9	n=10	n=10	n=9	n=10	n=10	n=9
ABC Scale	9.2000	−2.9000	9.0000	0.0279^*^	0.6004	0.1351	0.4757	0.4757	0.9865
FGA	2.9000	4.0000	3.5556	0.0002^*^	0.0006^*^	0.0095^*^	0.7308	0.7308	0.7308
MFIS	−5.0000	−6.5000	−13.3333	0.0835	0.0530	0.0175^*^	0.3762	0.7932	0.3762
MSSE	5.2000	1.7000	4.1111	0.0372^*^	0.3223	0.0618	0.7965	0.7965	0.7965
SF.36.mental	−2.0500	2.9667	11.8333	0.8389	0.0438^*^	0.0118^*^	0.0546	0.2112	0.0047^*^
SF.36.physical	3.2000	4.1556	21.7875	0.0849	0.0762	0.0073^*^	0.009^*^	0.8643	0.0090^*^
